# Access to care - an unmet need in headache management?

**DOI:** 10.1186/1129-2377-15-20

**Published:** 2014-04-17

**Authors:** Cristina Tassorelli, Ingemar Farm, Hilkka Kettinen, Elena Ruiz de la Torre, Srdjan Stretenovic, Wendy Thomas, Peter Vriezen, Leo Van Os, Dietmar Krause, Audrey Craven

**Affiliations:** 1 Headache Science Centre, C. Mondino National Neurological Institute, Pavia, Italy; 2 Department of Brain and Behaviour, University of Pavia, Pavia, Italy; 3 Swedish Migraine Association, Stockholm, Sweden; 4 Finnish Migraine Association, Helsinki, Finland; 5 Asociación Española Pacientes con Cefalea, Valencia, Spain; 6 Serbian Migraine Association, Belgrade, Serbia; 7 University Hospital KBC Zvezdara, Belgrade, Serbia; 8 Migraine Trust, London, UK; 9 Dutch Headache Patient Association, Rotterdam, The Netherlands; 10 German Green Cross, Tübingen, Germany; 11 Migraine Association of Ireland, Dublin, Ireland

**Keywords:** Headache disorders, Migraine, Healthcare, Management

## Abstract

Access to care for headache sufferers is not always simple. A survey conducted in a large number of members of lay associations point to the existence of multiple barriers to care for headache in several European countries. Patients usually discover the existence of specialized structures with a delay of several years after the onset of their headache. Furthermore, a relevant portion of them are not satisfied with the management of their disease, partly because of the poor efficacy of treatments and partly because of the difficulty to get in touch with the specialist. Headache disorders, and primary headaches in particular, represent an important issue in public health, because they are common, disabling and treatable. A joint effort is required from the relevant stakeholders (scientists, lay organizations, decision-makers, healthcare policymakers, and others) to improve the access to care for headache sufferers.

## Background

It is common knowledge that primary headaches, migraine in particular, are underdiagnosed and that, mostly because of this, migraine patients receive a suboptimal medical approach, even in developed countries [1,2].

Several explanations can be invoked for this phenomenon, i.e. low priority of headache in the list of interventions of national health system, insufficient awareness, inadequate preparation of GPs, limited availability of dedicated healthcare structures, stigma and many others [3-8].

In order to increase the attention of the problem of headache, besides and in addition to epidemiological and economic estimates, it seems important to take into considerations also the real life experience of patients. Since its foundation, in 2005, the European Headache Alliance (EHA), an umbrella organization of 21 national European Headache Patient Organisations (http://www.europeanheadachealliance.org) [9], has promoted several lay initiatives to increase headache awareness. During these activities, it has become increasingly evident that headache patients from several countries often meet various types of barriers when trying to access adequate health care for their condition. Therefore EHA undertook a more capillary survey in order to gather more detailed and representative information by patients themselves on the difficulty in accessing care for their headache.

This survey, named “Access to Care”, was conducted across several countries with the aim was to provide information directly from the headache sufferers to the policy makers, opinion leaders, researchers and physicians in order to provide information on a topic that is quite poorly explored and that it is vital for a better management of headache.

## Main text

For the survey, a simple questionnaire in English was devised (see Additional file 1) by the EHA Board, a structure formed by headache experts and headache sufferers. The questionnaire was circulated by e-mail to the representatives of EHA member lay associations in order to collect suggestions and comments. A final, consolidated version of the questionnaire in English was obtained, which took into consideration all the relevant comments. This version was circulated again to the representatives of EHA member lay associations for translation into their national language, when relevant.

The questionnaire was devised as to collect a minimum data set of anagraphic and clinical information, but it was totally anonymous, therefore neither privacy issues nor ethical regulations were applicable.

In the Spring of 2008, the questionnaire was posted for 3 months on the websites of the lay associations, asking their members to fill it in. Each lay association had to collect at least 50 consecutive questionnaires.

More than 1600 questionnaires were returned from 10 organisations in 9 European Countries: Finland, Germany, Ireland, Italy, Netherlands, Serbia, Spain, Sweden and United Kingdom.

Five years later, in the Spring of 2013, the questionnaire was posted again on the website sof the lay associations for 3 months. More than 1900 questionnaires were collected from 8 organizations in 8 European Countries: France, Germany, Ireland, Italy, Netherlands, Spain, Sweden and United Kingdom.

Responders were mostly women, with a distribution that was quite constant across countries. Most of responders were in their most active period of life (91% were aged from 20 to 60 years) and the majority suffered from migraine (see Additional file 2 for more details).

Most respondents (65%) were regularly seeing a health professional because of their headache, which was represented by the general practitioner or by a neurologist. Only a minority of subjects was being followed by a headache specialist.

Overall, nearly 60% of patients in the first edition of the survey and 48% in the second edition were not satisfied with their treatment, although the percentage tended to vary from country to country (Tables 1 and 2) with a minimum in Italy (43%) and a maximum in Spain (81%). Reasons for not being satisfied with treatment were, in order of frequency, ineffectiveness of prescribed drugs, difficulty in accessing a specialist, difficulty in having a follow-up appointment close in time and insufficient explanations.

**Table 1:** Percentage of headache sufferers who were or were not satisfied with the management of their headache in the survey run in 2008

**Countries**	**Total Responders N.**	**Satisfied%**	**Not satisfied%**
Finland	363	58	42
Germany	561	38	62
Ireland	60	56	44
Italy	82	42	58
Netherlands	98	43	57
Serbia	NA	-	-
Spain	137	19	81
Sweden	173	33	67
UK	68	47	53

**Table 2:** Percentage of headache sufferers who were or were not satisfied with the management of their headache in the survey run in 2013

**Countries**	**Total Responders N.**	**Satisfied%**	**Not satisfied%**
France	204	58	42
Germany	191	68	32
Ireland	174	59	41
Italy	221	44	56
Netherlands	69	52	48
Spain	278	19	81
Sweden	549	31	69
UK	190	34	67

A large portion of responders were unaware of the existence of specialist headache centres in their country (Tables 3 and 4), although, also in this case, a quite high variability was observed. It is noteworthy that the subjects who were aware of the existence of headache centres, they became so after a mean latency of 11 years from the onset of their headache in the first survey and with a latency of 9 years in the second survey. The main source of information was represented in both surveys by media, followed by medical experts and by patient organisations.

**Table 3:** Percentage distribution across countries of patients who were or were not aware of the existence of headache centres in their country in the survey run in 2008

**Countries**	**Total Responders N.**	**Aware%**	**Not aware%**
Finland	NA	-	-
Germany	561	45	55
Ireland	58	88	12
Italy	82	100	0
Netherlands	96	90	10
Serbia	100	65	35
Spain	136	23	77
Sweden	170	62	38
UK	67	57	43

**Table 4:** Percentage distribution across countries of patients who were or were not aware of the existence of headache centres in their country in the survey run in 2013

**Countries**	**Total Responders N.**	**Aware%**	**Not aware%**
France	204	79	21
Germany	191	87	13
Ireland	174	55	45
Italy	221	96	4
Netherlands	69	94	6
Spain	278	19	81
Sweden	549	46	54
UK	190	49	51

A second run of the survey was conducted in the Spring of 2013, with the same methodology used in the first run. Quite disappointingly, the results we obtained confirmed the situation illustrated above (see Additional file 2).

## Discussion

The present data provide information from a large number of headache sufferers across Europe. These findings reflect the direct experience of these subjects in accessing care for solving their headache problem. Obviously, this data is not the result of a strict methodologically-conducted epidemiological survey, nonetheless, for the specific declared aim of this commentary, they clearly point to a specific, unmet need.

The data were collected by a population that reflects quite well the general “headache” population, as described in the scientific literature, in terms of female prevalence and age distribution and their importance seems further corroborated by the fact that the barriers to care for headache turned out to be present and quite similar in all the different countries that participated in the survey.

Of course, the fact that the survey is based on members of lay organizations, i.e. a selected population, may represent a bias of the survey. However for the purposes of the present commentary - declaredly to draw the attention of stakeholders on the barriers that headache patients meet in their quest for care - we feel that collecting the view of members of a lay national organization may be of importance because these subjects theoretically represent a population of individuals that have better access to information, and therefore are probably better equipped than the general population to find a way toward proper care access. Furthermore, they obviously represent individuals who were affected by the headache enough to search and engage in an external support (the lay organization). So it seems that barriers to care might be even worse in the real life condition, when considering also the subjects who do not seek medical attention. At present we do not have data to substantiate the latter sentence, but we hope that the present commentary will stimulate adequate research on this vital issue for the battle against headache.

Along this line of reasoning, the fact that most subjects were not satisfied with the management of their disorder might actually be a biased result, related to the fact that lay associations may collect patients who seek further help because of a resistance or intolerance to commonly prescribed treatments. However, the fact that it takes many years for the subjects to become aware of the existence of headache centres does acquire here an inescapable relevance, exactly because it comes from subjects who seek information and help in multiple ways. Along the same line of reasoning, it seems relevant that only a small percentage of headache sufferers were followed in specialized centres, which again points to the importance of improving the quality and the offer of adequate healthcare solutions for these subjects.

## Conclusions

The present results point to the existence of multiple barriers to care for headache in several European countries and underline the pressing need of improving the access to care for headache sufferers in many ways:

– Information and education of patients and of doctors;

– Definition and dissemination of simple and shared models of care for headache;

– Increased availability of specialized centres;

– Increased awareness and consideration of headache by the policy makers.

The relative stability of most of the variables over five years, notwithstanding the advances in the scientific field and the effort of the lay organizations (nationally and at the European level), strongly suggests the need for a more coordinated and collaborative effort between lay associations, scientific bodies and health policy makers in order to improve the quality of care for headache disorders and the access to it for sufferers.

## Competing interests

The authors declare that they have no competing interests.

## Authors’ contributions

CT: conception of the survey, preparation of the questionnaire and of the methodology, collection of data from Italy, analysis of data, preparation of the commentary. EE: preparation of the questionnaire, collection of data from Sweden, revision of the manuscript. HK: preparation of the questionnaire, collection of data from Finland, revision of the manuscript. ERT: preparation of the questionnaire, collection of data from Spain, discussion of results, revision of the manuscript. SS: preparation of the questionnaire and of the methodology, collection of data from Serbia, discussion of results, revision of the manuscript. WT: preparation of the questionnaire and of the methodology, collection of data from UK, discussion of results, revision of the manuscript. PV: preparation of the questionnaire, collection of data from The Netherlands, first run of the survey, discussion of results, revision of the manuscript. LO: preparation of the questionnaire, collection of data from The Netherlands, second run of the survey, discussion of results, revision of the manuscript. DK: preparation of the questionnaire, collection of data from Germany (second run of the survey), discussion of results, revision of the manuscript. AC: conception of the survey, preparation of the questionnaire and of the methodology, collection of data from Ireland, analysis of data, discussion of results, revision of the manuscript. All authors read and approved the final manuscript.

## Supplementary Material

Additional file 1 Shows the questionnaire used for the survey. Click here for file

Additional file 2 Reports a selections of other findings from the two editions of the surveys (2008 and 2013). Click here for file
