# Supplementary material for: Access to care - an unmet need in headache management?
Source: J Headache Pain. 2014 Apr 17;15(1):20. doi: 10.1186/1129-2377-15-20 (PMC4021552; doi:10.1186/1129-2377-15-20)
Supplement: Additional file 1 — Shows the questionnaire used for the survey. [file 1129-2377-15-20-S1.pdf]

QUESTIONNAIRE FOR MEMBERS OF LAY HEADACHE ASSOCIATIONS

1) Please indicate your gender

- Female
- Male

2) Please indicate your age

- Below 20 years
- 21-40 years
- 41-60 years
- Above 61 years

3) Which of the following conditions is most applicable to you?

- Migraine
- Tension type headache
- Cluster headache
- Other Headache

4) How many headache days per month do you have?

5) Could you please indicate in which European country you live?

[Please select your country from the menu below]

6) If from a country with a public and private healthcare systems, please choose what type of patient you are:

- Public
- Private

7) How many years did you spend in school?

Please indicate the number: ___________

8) In your country, are headache diaries freely available in your national language?
[Please tick any of the statements that are true in your country]

- ○ Patient can easily find and download HEADACHE DIARIES from the Internet
- ○ Usually doctors will provide a paper copy of a headache diary
- ○ Sometimes
- ○ No
- ○ I am not sure

9) In your country, is access to reliable, accurate information on all aspects of headache* available?

- ○ Yes
- ○ No

If you answered yes,

9a) Where can you find it?
[Please tick any of the statements that are true in your country]

- ☐ From civil society organisations (such as patient organisations, etc).
- ☐ From medical professional organisations that specialise in head pain (such as neurologists' associations).
- ☐ The websites of hospitals/clinics.

Other source [please specify].

* Migraine and other headache disorders

10) Are you aware of the existence of programmes in your country to identify migraine in children who attend elementary/primary schools?

- Yes
- No

11) Are you aware of the existence of any national campaign in your country focused on increasing public awareness about headache* in the last 2 years?

- Yes
- No

12) Do you know about the existence of headache centres?

- Yes
- No

If you answered yes:

12a) How much time elapsed between the start of your headache* and the knowledge of a headache centre:

Please indicate the number in years:_______

12b) How did you find out about a headache centre:

- [ ] **Media (newspaper, TV etc.)**
- [ ] **Medical experts**
- [ ] **Leaflets, brochures etc.**
- [ ] **Other sufferers**
- [ ] **Patient organization**

- Relatives, friends, neighbours etc.
- Other

13) In your experience, have you had the right to choose which specialist doctor you see and which hospital you attend?

- Yes
- No

14) In your experience, can patients have easy access to information that allows them to see which hospitals/clinics are best for head pain (such information might, for instance, be about treatment results, complication rates, etc).

- Yes.
- Sometimes/it depends
- No
- I am not sure

15) Have you been referred to a headache* specialist?

- Yes
- No

If you answered yes:

15a) How were you referred?

- Referral from GP
- Direct Access (without referral)
- Other [please specify below]

15b) How long did you wait for the appointment with the specialist?

[technical comment: scroll down list, only 1 option]

☐ Less than three months.

☐ MORE THAN three months.

16) In your country, do you know if there are nurses with a SPECIAL COMPETENCE in headache*

○ Yes

○ No

○ I am not sure

If you answered yes, please specify where below

17) Do you regularly see a health professional for treatment?

○ Yes

○ No

If you answered yes,

17a) Who do you regularly consult?

☐ **General Practitioner**

☐ **Neurologist**

☐ **Headache Clinic staff**

☐ **Pharmacist**

☐ **Other (please specify below):**

18) What type of acute medication do you take?

[Please tick any classes that applies]

- ○ Over the counter painkillers
- ○ Prescription analgesics
- ○ Triptans or Ergotamine
- ○ Nothing

19) Do you take prophylactic medications?

- ○ Yes
- ○ No

If you answered yes,

19a) What length of time elapsed between first consultation with Health Professional and being prescribed preventative medication?

Please indicate in years:_____

20) Are you satisfied with the management and treatment of your headache condition?

- ○ Yes
- ○ No

If you answered no:

20a) What is the reason?

[Please tick any option that applies]

☐ **Ineffective treatment**

☐ **Explanation of treatment is insufficient**

☐ **Difficulty in getting to a headache specialist**

☐ **Next appointment too long ahead**

☐ **Other reasons, please specify:**
