# Supplementary material for: Access to care - an unmet need in headache management?
Source: J Headache Pain. 2014 Apr 17;15(1):20. doi: 10.1186/1129-2377-15-20 (PMC4021552; doi:10.1186/1129-2377-15-20)
Supplement: Additional file 2 — Reports a selections of other findings from the two editions of the surveys (2008 and 2013). [file 1129-2377-15-20-S2.docx]

NUMBER OF QUESTIONNAIRES RECEIVED IN SURVEY I: 1634

PARTICIPATING COUNTRIES: *Finland, Germany, Ireland, Italy, The Netherlands,*

*Serbia, Spain, Sweden, UK*

NUMBER OF QUESTIONNAIRES RECEIVED IN SURVEY I: 1935

PARTICIPATING COUNTRIES: *France*, *Ireland, Finland,* *Italy,* *Lithuania*, *Romania*, *Serbia*, *Spain*, *Sweden*, *The Netherlands*, *UK*

Sex distribution of responders to both surveys

|  | Total N. | Women  % | Men  % |
| --- | --- | --- | --- |
| Survey I | 1634 | 83.8 | 16.2 |
| Survey II | 1935 | 81.8 | 18.2 |

Diagnoses reported by responders

|  | Migraine  N % | | Tension-type headache  N % | | Cluster Headache  N % | | Other headaches  N % | |
| --- | --- | --- | --- | --- | --- | --- | --- | --- |
| Survey I | 1112 | 67.9 | 326 | 20.1 | 98 | 6.0 | 98 | 6.0 |
| Survey II | 1345 | 69.5 | 346 | 17.9 | 142 | 7.3 | 102 | 5.3 |

Health professional figures consulted by the responders

|  | Total N. of subjects who consulted a health professional | General practitioner  N % | | Neurologist  N % | | Headache  Specialist  N % | | Pharmacist  N % | | Other  N % | |
| --- | --- | --- | --- | --- | --- | --- | --- | --- | --- | --- | --- |
| Survey I | 1045 | 387 | 37 | 313 | 30 | 84 | 8 | 63 | 6 | 198 | 19 |
| Survey II | 1179 | 389 | 33 | 472 | 40 | 212 | 18 | 12 | 1 | 94 | 8 |

Satisfaction with the management of headache

|  | Total N. | Satisfied  % | Not satisfied  % |
| --- | --- | --- | --- |
| Survey I | 1634 | 41 | 59 |
| Survey II | 1935 | 52 | 48 |

Top four reasons for not beeing satisfied with the management of headache (possible multiple choices)

|  | Total N. of subjects who were not saitsfied with management | Ineffectiveness of drugs  N % | | Difficulty  in getting  to a headache  specialist  N % | | Difficulty in having a follow-up appointment close in time  N % | | Insufficient explanations  N %) | |
| --- | --- | --- | --- | --- | --- | --- | --- | --- | --- |
| Survey I | 964 | 540 | 56.0 | 291 | 30.2 | 142 | 14.7 | 137 | 14.2 |
| Survey II | 929 | 401 | 43.2 | 201 | 21.7 | 129 | 13.9 | 154 | 16.6 |
